# Supplementary material for: k-mer manifold approximation and projection for visualizing DNA sequences
Source: Genome Res. 2025 May;35(5):1234–46. doi: 10.1101/gr.279458.124 (PMC12047656; doi:10.1101/gr.279458.124)
Supplement: Supplement 8 [file Supplemental_Data_1.zip › Supplemental_Data_1/ELF5_TGCCGC20NCG_V_4/ELF5_TGCCGC20NCG_V_4_KMAP.pdf]

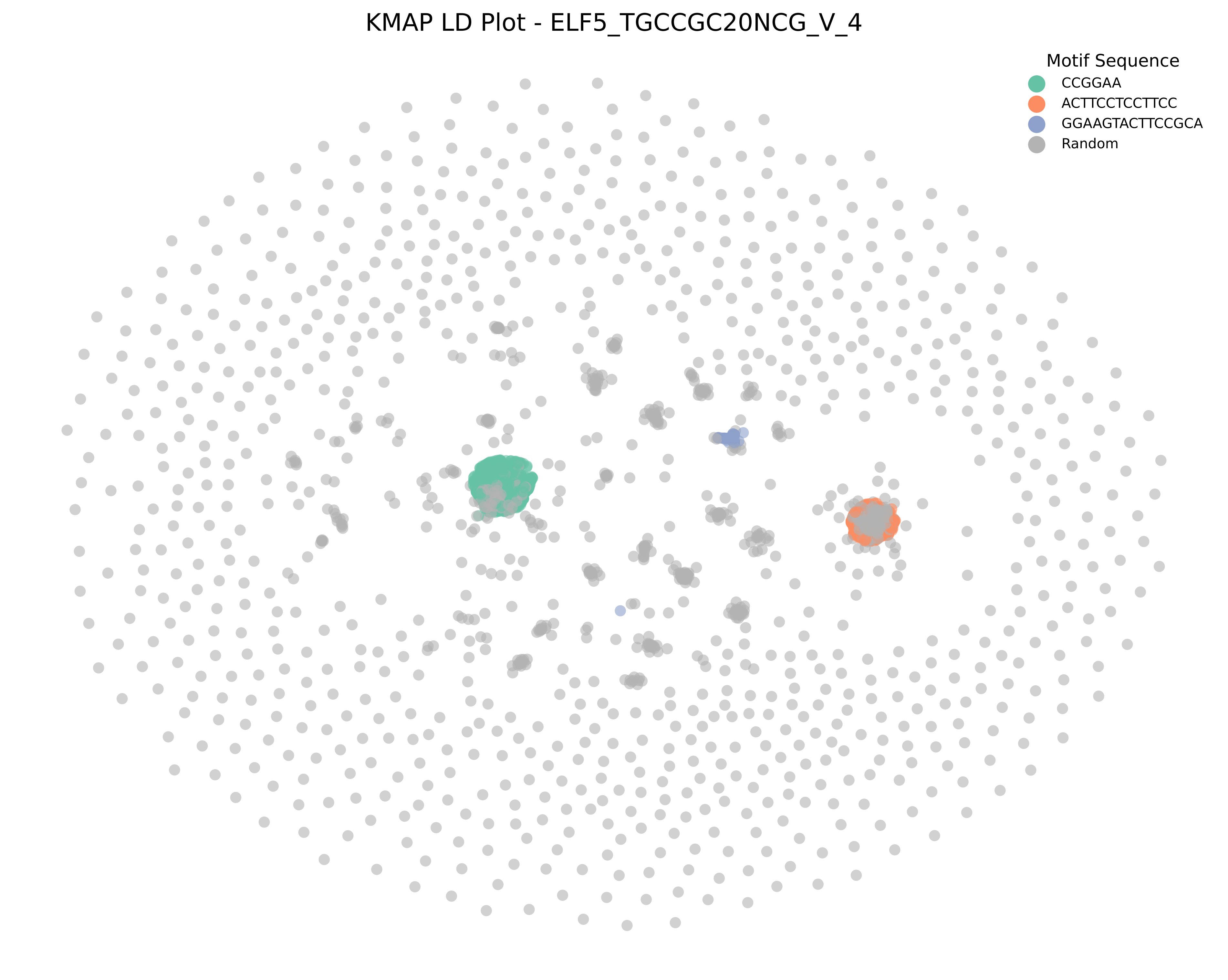
KMAP LD Plot - ELF5_TGCCGC20NCG_V_4
Motif Sequence
CCGGAA
ACTTCCTCCTTCC
GGAAGTACTTCCGCA
Random
